# Alcohol induced behavioral and immune perturbations are attenuated by activation of CB2 cannabinoid receptors

**DOI:** 10.3389/adar.2023.11602

**Published:** 2023-12-19

**Authors:** Aaliyah Roberts, Mahli Christian, Lizbeth Nivar Dilone, Natania Nelson, Mark Joseph Endrino, Adam Kneebone, Shymaa Embaby, Justin Fernandez, Qing-Rong Liu, Emmanuel S. Onaivi, Berhanu Geresu Kibret

**Affiliations:** ^1^ Department of Biology, College of Science and Health, William Paterson University, Wayne, NJ, United States; ^2^ Laboratory of Clinical Investigation, National Institute on Aging, National Institutes of Health, Baltimore, MD, United States

**Keywords:** eCBome, ECS, alcohol, inflammation, CB2Rs

## Abstract

The endocannabinoidome (eCBome) is the expanded endocannabinoid system (ECS) and studies show that there is a link between this system and how it modulates alcohol induced neuroinflammation. Using conditional knockout (cKO) mice with selective deletion of cannabinoid type 2 receptors (CB2Rs) in dopamine neurons (DAT-*Cnr2*) and in microglia (Cx3Cr1-*Cnr2*), we investigated how CB2Rs modulate behavioral and neuroinflammation induced by alcohol. Behavioral tests including locomotor and wheel running activity, rotarod performance test, and alcohol preference tests were used to evaluate behavioral changes induced by alcohol. Using ELISA assay, we investigated the level of pro-inflammatory cytokines, tumor necrosis factor-α (TNF-α), interleukin-6 (IL-6), interleukin-1α (IL-1α), and interleukin-1β (IL-1β) in the hippocampus of mice. The findings demonstrated that locomotor activity, wheel running, and rotarod performance activities were significantly affected by cell-type specific deletion of CB2Rs in dopamine neurons and microglia. The non-selective CB2R agonist, WIN 55,212-2, reduced alcohol preference in the wild type and cell-type specific CB2R cKO mice. In addition, the result showed that cell-type specific deletion of CB2Rs *per se* and administration of alcohol to CB2R cKO mice increased the expression of proinflammatory cytokines in the hippocampus. These findings suggest the involvement of CB2Rs in modulating behavioral and immune alterations induced by alcohol.

## Introduction

The characterization of additional lipid mediators, enzymes and receptors, has led to the discovery of an expanded endocannabinoid system (ECS) called the endocannabinoidome (eCBome) [1]. The ECS is composed of two canonical cannabinoid receptors (CBRs); cannabinoid type 1 receptors (CB1Rs) and cannabinoid type 2 receptors (CB2Rs), endocannabinoids (eCBs) and enzymes responsible for the synthesis and degradation of eCBs [2, 3]. While cannabinoids represent a group of substances that share the common property of binding with cannabinoid receptors (CBRs), only two substances, arachidonoyl ethanolamide (anandamide) and 2-arachidonoyl glycerol, are considered primary eCBs [4–6]. CB1Rs, which are expressed in the hippocampus, neocortex, cerebellum, and basal ganglia nuclei, are the most abundant GPCRs in the brain [3]. CB2Rs are found in abundance in the periphery and predominantly in organs with immune function [7–9]. Contrary to the previous notion that CB2Rs were absent in brain [9–11], a growing body of evidence now demonstrates CB2R expression in microglia, and neurons in the hippocampus, striatum and brain stem [12, 13]. There has been continuous debate and controversy about the expression of functional neuronal CB2Rs, however, following our discovery of the presence and functional expression of CB2Rs in brain [14–17], other studies have overwhelming confirmed that functional CB2Rs are present in neurons and are regulated by drugs of abuse [18–21].

Chronic alcohol consumption, through abnormal brain circuits, can cause neuronal damage, behavioral alterations, and neuroinflammation that are characterized by an enhanced release of pro-inflammatory cytokines called cytokine storm [22–24]. Recent preclinical reports suggest that enhanced innate immune system signaling increases consumption of alcohol [25]. Studies also indicated that CB2R activation has been shown to inhibit neuroinflammation, attenuate neuronal tissue damage, and drive neurogenesis [26, 27]. We hypothesized that CB2Rs can play a role in preventing alcohol induced behavioral and neuroimmune changes in mice. We addressed this question by investigating the roles of dopamine neuron and microglia CB2Rs using DAT-*Cnr2*, Cx3cr1-*Cnr2* cKO, and wild type (WT) control mice in modulating behavioral and neuroimmune alterations induced by the effects of alcohol.

## Materials and methods

### Animals

In this study, we employed DAT-*Cnr2* and Cx3Cr1-*Cnr2* cKO mice which are created in our lab [28]. The mice were generated through a breeding approach involving *Cnr2*-floxed mice and DAT-*Cre* and Cx3-*Cre* mice. We confirmed the specific deletion of CB2Rs in dopamine cells and microglia in homozygous cKO mice through genotyping and RNAscope *in situ* hybridization, while no deletion occurred in the WT mice. The experiments were conducted on adult male mice weighing between 20 g and 30 g, all bred in the mouse laboratory at William Paterson University of New Jersey. These mice were kept under controlled conditions, including room temperature (25°C ± 2°C), a 12:12 h light-dark cycle, and *ad libitum* access to food and water. Our study adhered to the guidelines in the Guide for the Care and Use of Laboratory Animals and received approval from the William Paterson University Animal Care and Use Committee (IACUC).

### Drugs and administration

Absolute ethanol was purchased from Pharmaco-AAper in Bristol, PA. 8% of the absolute alcohol was mixed with distilled water and administered as 0.8 g/kg dose into the peritoneum (i.p.) at a volume of 10 mL/kg body weight. The non-selective cannabinoid receptor agonist, WIN55,212-2 (WIN), was purchased from Cayman Chemical Co. located in Ann Arbor, MI. After dissolving WIN in a mixture of DMSO, tween 80, and saline in a ratio of 1:2:7, a dosage of 3 mg/kg was administered. The doses of alcohol and WIN were determined based on previous research [21, 28–30]. Both alcohol and WIN were injected i.p. in a volume of 10 mL/kg body weight.

### Locomotor activity test

To evaluate total distance travelled in the activity box, the locomotor activity monitoring apparatus (ENV-510: Med Associates Inc., St. Albans, VT, USA) was utilized. Thirty minutes after acute alcohol injection, the animals were placed gently into separate test boxes (measuring 43.2 × 43.2 × 30.5 cm) that were connected to a computer. Total distance traveled by mice was recorded and analyzed over a 10-min period [21]. Prior to the test, the mice were given three consecutive days to freely explore the open field chambers for 10 min each day in order to acclimate to the environment.

### Wheel running activity test

The wheel running activity of the mice was observed using a spontaneous wheel-running monitor (Wahmann, Geo. H., Manufacturing Company, Baltimore, MD, USA) after 40 min of acute alcohol administration. Each mouse was placed in the monitor, and their wheel running behavior was tracked using auto-counters, which recorded the total number of revolutions made by each animal during the 10-min testing session [21].

### Rota rod performance test

Mice were placed on a stationary rota rod (AccuRotor Rotarod, AccuScan Instruments Inc.) by gently gripping their tails, positioning them away from the direction of rotation. To maintain balance, the mice had to walk forward on the rod. The rota rod was set at a height of 30 cm above the ground and featured a rotating rod with a 3 cm diameter. The duration each mouse managed to stay on the rod for 180 s was recorded, excluding falls occurring within the initial 5 s due to improper placement by the experimenter [21]. A soft padded surface was positioned at the base of the apparatus to cushion any falls.

### Alcohol preference test

For preference testing, individually housed mice (N = 10 mice per group) were used. Throughout a 24 h period, the mice had access to two conical tubes with a drinking spout attached filled with water. In order to institute a baseline, both tubes were initially filled with 40 mL of water and placed above the cages for three consecutive days. During the preference measurement phase, one of the tubes was replaced with a solution containing 8% alcohol. The amount of alcohol consumed by each animal was recorded over five consecutive days between 10 and 11 AM. To ensure unbiased positioning, the placement of the tubes within the various cages was randomized with regard to the side of the cages they were placed on. In all experiments, the ratio of alcohol to water consumed, and the total fluid consumption, were calculated to obtain a preference ratio. Additionally, half of the animals in each group (N = 5) were injected with WIN daily for five consecutive days. The alcohol preference ratio was determined by dividing the amount of alcohol consumed by the total fluid (alcohol + water) consumption [21].

### Cytokine assay

Mice involved in the acute behavioral experiments were continuously administered either the vehicle or alcohol for seven consecutive days. On the eighth day, the mice were decapitated, and their brains were removed from the skull. To aid dissection, the brains were promptly frozen in liquid nitrogen. Specific brain regions containing the hippocampus were dissected and placed in cell lysis buffer. Using an ultrasonic homogenizer, the tissue was homogenized. The resulting homogenates were then centrifuged at 10,000 RPM for 5 min to separate the tissue debris. Samples of the resulting supernatants were collected and, after determining the protein concentration, frozen and stored at −80°C until needed for cytokine analysis. To profile the expression of IL-1α (interleukin-1α), IL-1β (interleukin-1β), IL-6 (interleukin-6), and TNF-α (tumor necrosis factor-α), a Mouse Inflammation ELISA Strip kit (Signosis, Sunnyvale CA, USA) was employed. In brief, 100 μL of the diluted cell lysate sample was added to wells coated with a specific primary antibody against each cytokine. After incubation for 1 h at room temperature, the wells were aspirated and washed three times with 200 μL of assay wash buffer. Subsequently, 100 μL of a biotin-labeled antibody mixture was added to each well and incubated for 1 h at room temperature. The wells were again aspirated and washed three times with 200 μL of assay wash buffer. Then, 100 μL of streptavidin-HRP conjugate was added to each well and incubated for 45 min at room temperature. Following aspiration and another round of washes, 100 μL of substrate was added and incubated for 10 min, followed by the addition of 50 μL of stop solution to each well. The optical density of each well was measured using a microplate reader at 450 nm [21].

### Statistical analysis

Data are presented as mean ± SEM. Sigma Plot 12.0 statistical program was used. Prior to performing the tests, we conducted a normality test (Shapiro-Wilk) to verify the distribution of the data. The statistical analysis was performed by the two-way analysis of variance (ANOVA). Post hoc comparisons of means were carried out with Tukey’s test for multiple comparisons when appropriate. We used two-way ANOVA for the analysis of behavioral and cytokine assay data. Data from the alcohol preference study were analyzed by using repeated measures two-way ANOVA. The confidence limit of *p* < 0.05 was considered statistically significant. One of the factors of the ANOVA was the genotype (DAT-*Cnr2*, Cx3Cr1-*Cnr2* or WT mice) and the other factor was treatment groups (vehicle or alcohol).

## Results

### Brain CB2Rs modifies locomotor activity induced by alcohol

**FIGURE 1:**
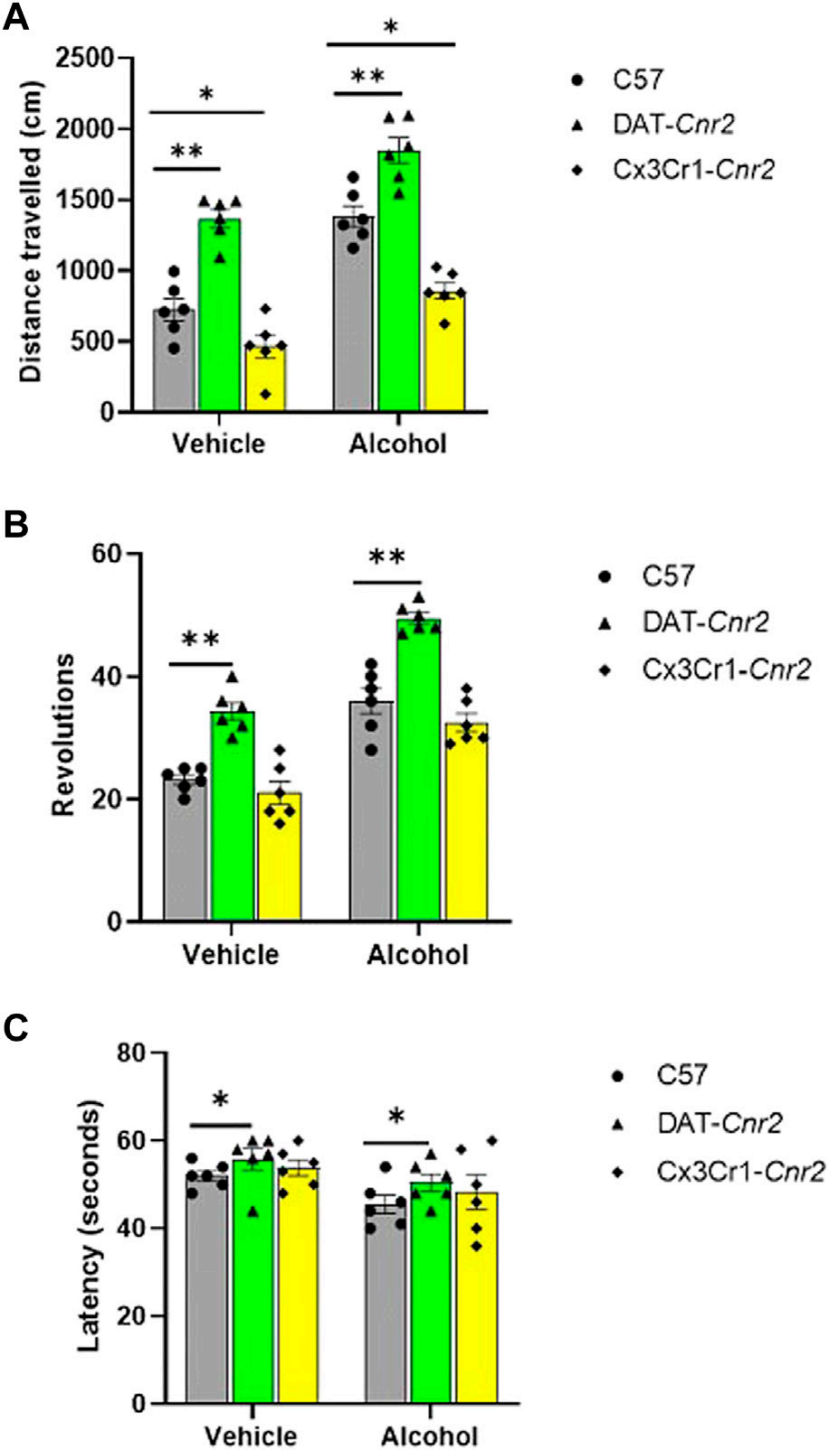
Acute effect of alcohol (0.8 g/kg) on distance travelled (centimeters) in the activity monitor apparatus **(A)**, on the absolute number of revolutions in the wheel-running test **(B)** and on fall latency (seconds) in the rotarod test **(C)** in WT, DAT-*Cnr2*, Cx3Cr1-*Cnr2* mice. Values are mean ± SEM (*n* = 6 in each group). Statistical analysis was done using Two-way ANOVA test. **p < 0.01, *p < 0.05 compared to C57-WT group.

We evaluated acute motor activity in C57, DAT-*Cnr2*, and Cx3Cr1-*Cnr2* mice following the administration of 8% alcohol using an activity monitor apparatus. The results showed significant main effects for both treatment and genotype (*F*_1, 30_ = 70.30, *p* < 0.001 and *F*_2, 30_ = 81.53, *p* < 0.001, respectively), as well as a significant interaction between treatment and genotype (*F*_2, 30_ = 16.22, *p* < 0.001). Post-hoc analysis using Tukey’s test for multiple comparisons revealed that alcohol administration significantly increased the total distance traveled in the activity box compared to the control group treated with vehicle. Interestingly, the results also indicated that specific deletion of CB2R in dopamine neurons (DAT-*Cnr2* cKO) enhanced alcohol-induced locomotor activity, with a statistically significant (*p* < 0.01) increase in the total distance traveled compared to WT mice. In contrast, the cell-type specific deletion of CB2R in microglia (Cx3Cr1-*Cnr2* cKO) reduced alcohol-induced locomotor activity, showing a statistically significant (*p* < 0.05) decrease in the total distance traveled compared to WT mice (Figure 1A).

### Dopamine and microglia specific deletions of CB2Rs enhance alcohol induced wheel-running activity

In this study, we investigated acute wheel running behavior in C57, DAT-*Cnr2*, and Cx3Cr1-*Cnr2* mice following the administration of 8% alcohol using a mechanical wheel running apparatus. The number of revolutions exhibited a significant association with the treatment groups (*F*_1, 30_ = 112.2, *p* < 0.001) and genotype (*F*_2, 30_ = 56.12, *p* < 0.001). Post-hoc analysis using Tukey’s test revealed that both the vehicle and alcohol treatment of DAT-*Cnr2* mice resulted in a significant (*p* < 0.01) increase in the absolute number of revolutions compared to the control group of WT mice (Figure 1B).

### Deletion of CB2R in dopamine neurons enhances alcohol induced reduction in fall latency

We examined the ability of mice to maintain their position on a rotating cylinder following the administration of 8% alcohol. We employed a constant speed rotarod apparatus for this assessment in C57, DAT-*Cnr2*, and Cx3Cr1-*Cnr2* mice. The results showed that cell type specific deletion of CB2R in dopamine neurons enhanced alcohol induced reduction in fall latency in the rotarod test of DAT-*Cnr2* mice, whereas this effect was not observed in the deletion of CB2R in microglia of Cx3Cr1-*Cnr2* mice. There was a significant main effect for both treatment and genotype (*F*_1, 30_ = 28.43, *p* < 0.001 and *F*_1, 30_ = 62.52, *p* < 0.001, respectively). Post-hoc analysis using Tukey’s test indicated a statistically significant (*p* < 0.05) increase in fall latency in DAT-*Cnr2* mice compared to the WT controls. However, the cell-type specific deletion of CB2R in microglia did not affect the alcohol-induced changes in fall latency when compared to the WT controls (Figure 1C).

### WIN 55,212-2 reduces alcohol preference in the wild type and cell-type specific CB2R cKO mice

**FIGURE 2:**
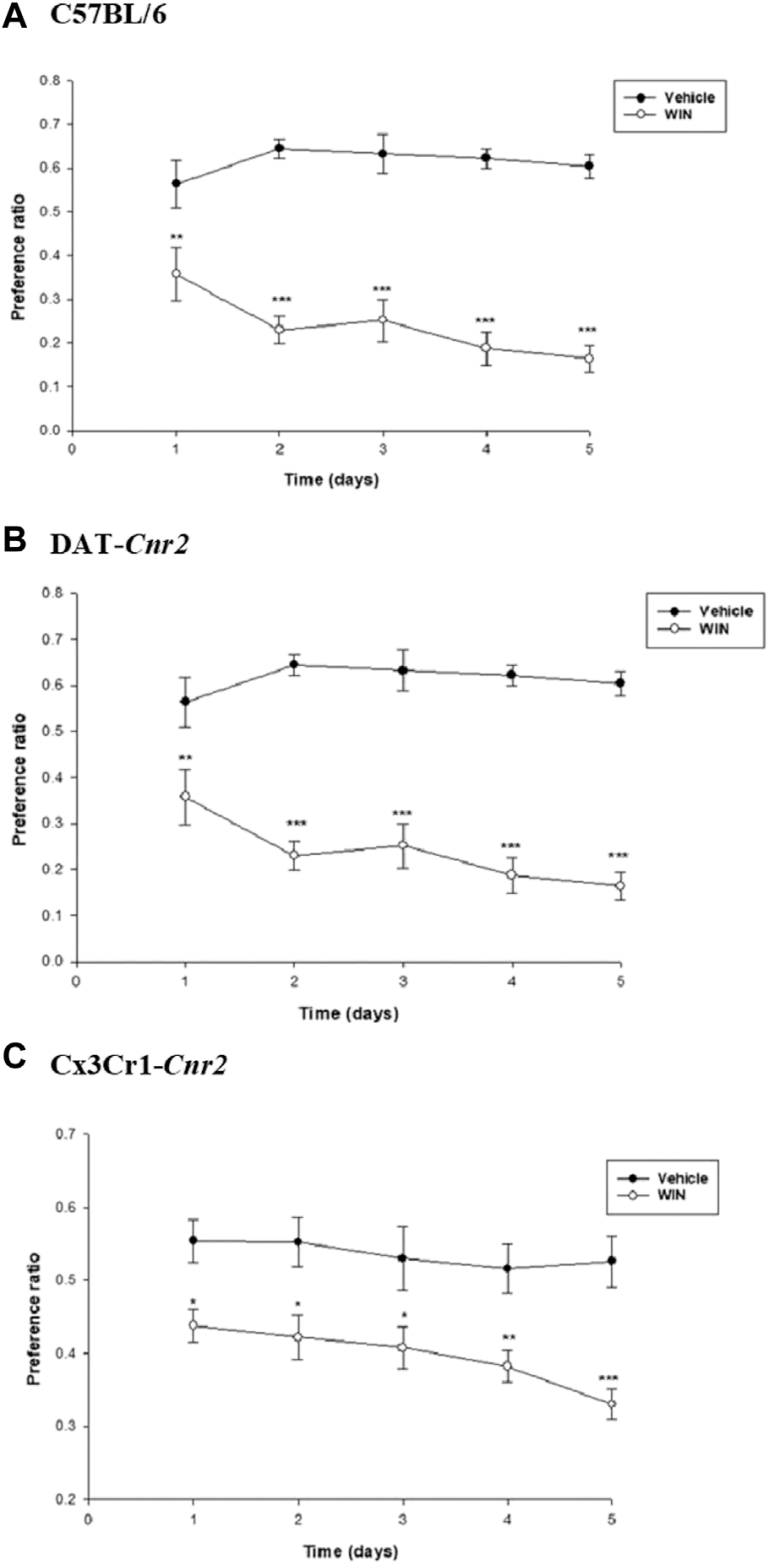
Role of WIN in alcohol preference. WIN significantly reduced alcohol preference in C57 wild type **(A)**, DAT-*Cnr2* **(B)** and Cx3Cr1-*Cnr2* **(C)** mice compared to vehicle treated controls. Values are mean ± SEM (*n* = 6 in each group). Statistical analysis was done using Repeated Measures Two-way ANOVA test. ***p < 0.001, **p < 0.01, *p < 0.05.

We further investigated the potential association between subacute treatment with WIN and alcohol preference. In WT mice, the results demonstrated a significant effect of both treatment and time on the alcohol preference ratio (*F*_1, 20_ = 79.229, *p* < 0.001 and *F*_4, 20_ = 3.172, *p* < 0.05, respectively), as well as a significant interaction between treatment and time (*F*_4, 20_ = 6.421, *p* < 0.05). Post hoc analysis revealed a significant (*p* < 0.01) reduction in alcohol preference in mice treated with WIN compared to the vehicle-treated controls (Figure 2A). Similarly, in DAT-*Cnr2* mice, there was a significant effect of both treatment and time on the alcohol preference ratio (*F*_1, 20_ = 233.855, *p* < 0.001 and *F*_4, 20_ = 4.956, *p* < 0.05, respectively), along with a significant interaction between treatment and time (*F*_4, 20_ = 9.042, *p* < 0.001). Post hoc analysis indicated a significant (*p* < 0.01) reduction in alcohol preference in mice treated with WIN compared to the vehicle-treated controls (Figure 2B). In Cx3Cr1-*Cnr2* mice, statistical analysis also revealed a significant effect of both treatment and time on the alcohol preference ratio (*F*_1, 20_ = 68.225, *p* < 0.001 and *F*_4, 20_ = 5.716, *p* < 0.05, respectively), as well as a significant interaction between treatment and time (*F*_4, 20_ = 2.812, *p* < 0.05). Post hoc analysis demonstrated a significant (*p* < 0.05) reduction in alcohol preference in mice treated with WIN compared to the vehicle-treated controls (Figure 2C).

### CB2Rs reduce alcohol induced increase in pro-inflammatory cytokines in mice hippocampus

**FIGURE 3:**
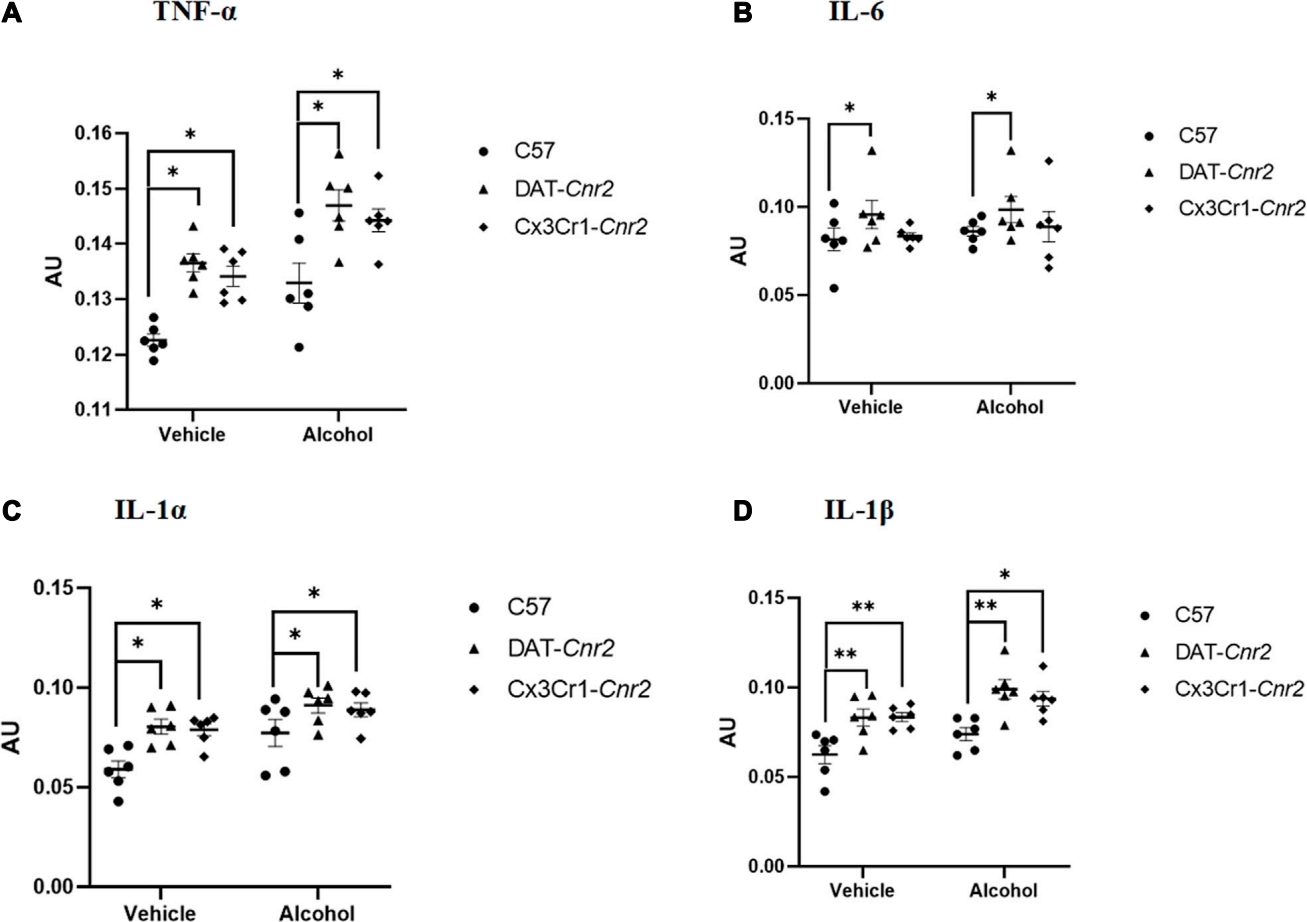
Measures of the levels of proinflammatory cytokines TNF-α **(A)**, IL-6 **(B)**, IL-1α **(C)** and IL-1β **(D)** in the hippocampus of mice (WT, DAT-*Cnr2*, Cx3Cr1-*Cnr2*) after seven consecutive days of sub-acute treatment with vehicle or alcohol (0.8 g/kg). Statistical analysis was done using Two-way ANOVA followed by Tukey’s multiple comparisons test. Values are mean ± SEM (*n* = 4 in each group). **p < 0.01, *p < 0.05. AU – absorbance unit.

The result form the cytokine study showed both treatment and genotype significantly affected the expression of TNF-α [treatment effect: *F*_1, 30_ = 29.33, *p* < 0.001; genotype effect: *F*_2, 30_ = 20.51, *p* < 0.001; treatment X genotype interaction: *F*_2, 30_ = 5.43, *p* < 0.05] and IL-1β [treatment effect: *F*_1, 30_ = 12.27, *p* < 0.001; genotype effect: *F*_2, 30_ = 16.43, *p* < 0.001; treatment X genotype interaction: *F*_2, 30_ = 9.62, *p* < 0.01]. Compared to the WT controls, Tukey’s test revealed that there was statistically significant increase in the levels of TNF-α and IL-β, as evidenced by enhanced absorbance values, in DAT-*Cnr2* and Cx3Cr1-*Cnr2* mice treated with alcohol (Figures 3A–D).

## Discussion

Due to the neuro-immune functioning associated with the reward pathway, recently, there is an increasing interest and attention on CB2Rs as a target for the treatment of drug addiction [31–34]. The aim of this study was to examine the effect of genetic and pharmacological modulation, using the non-selective CBR agonist WIN 55, 212-2, of CB2Rs on behavior and neuro-immune changes induced by alcohol. The results demonstrate that cell-type specific deletion of CB2Rs in dopamine neurons and microglia significantly altered locomotor activity, and wheel running activity, and on the rota rod performance test. The results also revealed that cell-type specific deletion of CB2Rs enhanced alcohol-induced inflammation. In addition, pharmacologic activation of CB2Rs using WIN 55, 212-2 reduced alcohol preference.

The results of the current study support our earlier finding that CB2Rs acts as a “brake” on dopamine neurons’ ability to activate the locomotor system and that its deletion in DAT-*Cnr2* cKO mice improves psychomotor behavior [21, 28, 35]. The observation that deletion of CB2Rs in DA neurons resulted in enhanced spontaneous motor activity reinforces the notion that CB2R mediates inhibition of spontaneous movement via modulation of the dopamine system, probably through reduction of neuronal firing frequency [36]. However, in contrast to the DAT-*Cnr2* mice, Cx3Cr1-*Cnr2* mice showed a reduction in locomotor activity compared to the wild type controls. *In vitro* and *in vivo* studies demonstrated that activation of CB2R decreases inflammation and protect neurons from degeneration [26, 27]. In this study, the hypolocomotion observed in the Cx3Cr1-*Cnr2* mice might be due to lack of the neuroprotective effects of CB2Rs from neurodegeneration.

Alcohol dose, route of administration, and mouse strain all have an impact on how alcohol affects locomotor activity in mice. In this work, we discovered that locomotor activity was increased in both the wild-type and genetically modified mice after sub-acute i.p. administration of 8% alcohol. Previous research have shown that alcohol enhances locomotor activity and locomotor sensitization [37–42], which is consistent with the findings of the present investigation.

Our investigation into the subacute effects of the WIN compound on alcohol preference revealed that it greatly decreased alcohol intake in DAT-*Cnr2* and Cx3Cr1-*Cnr2* cKO mice, providing one piece of support for the idea that CB2Rs are involved in the behavioral effects of alcohol. In our previous study we showed that the DAT-*Cnr2* cKO mice consumed less alcohol than wild type mice with and without the stress, suggesting that the deletion of CB2Rs in DA neurons contributed to the reduction in alcohol consumption and preference [28]. Studies showed contradicting result on the effect of CB2Rs on ethanol intake. Some reported that a naturally available full-agonist of CB2Rs, beta-caryophyllene (BCP) lowered ethanol intake in the two bottle paradigm in mutant *Cnr2* ^−/−^ mice [20, 43] whereas, others reported that sub-chronic injection of JWH015 enhanced alcohol intake in mice [44, 45]. The variation in response might be due to different factors such as concentration and route of administration of ethanol, duration of exposure, strain of animal and the animal model used in the experiment. However, accumulating data support a role of CB2Rs in modulating the addictive effects of alcohol indicating that CB2Rs might be targeted in the treatment of behavioral impairment induced by alcohol consumption.

Alcohol causes organ damage that affects the liver, cardiovascular system, and brain. This organ damage is characterized by inflammation and altered innate immune responses [46–48]. Chronic alcohol consumption results in neuroinflammation [49] and neurodegeneration in humans as well as animal models, as evidenced by increased expression of MCP-1, TNF-α, IL-1β and caspase-3 in the brain [48, 50, 51]. The hippocampus has been repeatedly affected by the neuroimmune dysregulations induced by alcohol [52]. Here we report that cell-type specific deletion of CB2Rs *per se* and administration of alcohol to CB2R cKO mice increased the expression of proinflammatory cytokines TNF-α, IL-6, IL-1α and IL-1β in the hippocampus of mice, which is an evidence for the neuroprotective role of CB2Rs. The use of CB2R ligands in the neuroprotective and anti-inflammatory activity linked to neuropsychiatric and neurodegenerative disorders is based on the fact that CB2Rs expression is increased during injury and inflammation, with their upregulation during CNS disorders providing a basis and focus of attention [33, 53]. Studies showed that the activation of CB2R is related to decreases in pro-inflammatory cytokines (TNF-α, interferon gamma (IFN-ɣ), IL-1, IL-2, IL-6 or IL-12) [21, 54–56]. The outcome of our current investigation points to a critical role for CB2Rs and neuroinflammatory processes in alcohol-related neurobiological and behavioral changes. However, it should be noted that complete loss of the anti-inflammatory CX3CR1 receptor in homozygous mice is a potential confounder since this receptor is important for sustaining normal microglia function and lack of CX3CR1 reportedly results neurotoxic microglia phenotype. To prevent alcohol-induced neuroinflammation and related brain dysfunctions, pharmacological regulation of CB2Rs may be a focus. In summary, cell-type specific deletion of CB2Rs enhances psychomotor activity and increases the level of proinflammatory cytokines in the hippocampus. In addition, pharmacologic modification of CB2Rs using the WIN 55,212-2 compound reduced alcohol consumption in mice compared to vehicle. However, more studies are required to provide additional molecular and cellular mechanisms associated with neuro-immuno-eCB modulation of the effects of alcohol and CB2Rs in autoimmune disorders.

## Data Availability

The original contributions presented in the study are included in the article/supplementary material, further inquiries can be directed to the corresponding authors.
